# Exploring the subjective role of diet for people living with severe mental illness: insights from a biopsychosocial perspective

**DOI:** 10.1192/j.eurpsy.2025.601

**Published:** 2025-08-26

**Authors:** A. S. Mueller-Stierlin, R. Hiltensperger, S. Mörkl, S. Teasdale

**Affiliations:** 1 Institute for Epidemiology and Medical Biometry, University of Ulm, Ulm, Germany; 2 Klinische Abteilung für medizinische Psychologie, Psychosomatik und Psychotherapie, Medizinische Universität Graz, Graz, Austria; 3 University of New South Wales, Sydney, Australia

## Abstract

**Introduction:**

People living with severe mental illness (SMI) face a life expectancy reduction of 10 to 20 years, often due to physical comorbidities. In addition to medication side effects, unhealthy lifestyle choices may contribute to this disparity.

**Objectives:**

Understanding the experiences and views of people living with SMI regarding diet is essential in addressing these challenges.

**Methods:**

To explore the role of nutrition and its determinants within a biopsychosocial framework, 28 semi-structured interviews were conducted with service users living in Germany, Austria, and Australia. A generic thematic analysis was applied to uncover key themes around implications of dietary behavior and its determinants.

**Results:**

Both positive and negative effects of diet were reported. A prominent theme was the mental strain related to body weight, which contributed to feelings of guilt and experiences of stigma. Numerous biological, psychological, and social factors were identified as influencing dietary choices and behaviors. Many participants expressed a desire for greater support in achieving dietary balance and breaking the vicious cycle between diet and mental health.

**Conclusions:**

From the viewpoint of people living with SMI, dietary interventions should be more integrated into mental health care. Psychosocial aspects, such as the emotional impact of eating, are as important as biological factors like nutrient intake, emphasizing the need for a holistic approach to addressing diet in mental health care.

**Disclosure of Interest:**

None Declared

